# Exciting strain‐level resolution studies of the food microbiome

**DOI:** 10.1111/1751-7915.12593

**Published:** 2017-01-03

**Authors:** Danilo Ercolini

**Affiliations:** ^1^ Department of Agricultural SciencesUniversity of Naples Federico II80055PorticiItaly

Attention to food quality and safety will never decrease. Thus, studies of food microbiology and food‐associated microbial ecology are always timely and scientifically relevant.

The ‘cultural’ evolution in food microbiology has shifted the focus from the traditional cultivation of food microbes to the consideration of food as a microbiologically dynamic matrix (Cocolin and Ercolini, 2015). Food can potentially host bacteria, yeasts, filamentous fungi and even viruses. These organisms do not simply inhabit food but also possibly contribute to food quality and safety, depending on what kind of activities and interactions are established in the conditions where foods are produced, stored and distributed. We experimented with a wide range of culture‐independent approaches to study microbial ecology. Today, we fortunately possess a huge toolbox of molecular biology and data analysis methods that we can use. Food microbiota is routinely studied with rRNA amplicon‐based high‐throughput sequencing approaches, which have good potential for food quality screening, monitoring population dynamics and meta‐analyses based on interactive databases of food microbiota (Ercolini, 2013; Parente *et al*., 2016). Additionally, metagenomics conducted with shotgun libraries can aid in the exploration of both taxonomic composition and functional diversity of microbial communities using a much larger amount of information. For example, in‐depth analysis of the cheese microbial maturation process and a better understanding of the metabolic activities and interactions within the cheese microbial community are possible with metagenomics. The cheese microbiome could serve as a simplified model for understanding microbial dynamics in even more complex environments (Wolfe *et al*., 2014). In addition, when metatranscriptomics data are available along with metagenomics data for the same sample, transcription rates for specific genes are obtainable. Therefore, *in situ* high‐throughput gene expression studies are readily available to explore functional variations in food microbial consortia (De Filippis *et al*., 2016).

Microbial communities can be studied at different levels of taxonomic resolution. However, identification and characterization of microbial strains, their genes and their functions are fundamental to the study of their role in food. This is traditionally accomplished with whole‐ or partial‐genome sequencing after strain isolation and cultivation *in vitro*, and many strains of food‐associated microbial species have been sequenced thus far.

The future of this research is the extraction of strain genome profiles from metagenomics data sets. Once nucleic acids are extracted from a food of interest, researchers can make shotgun libraries, obtain the metagenome by sequencing and then use up‐to‐date, challenging and rewarding collection of bioinformatics tools to study the genomes of the strains of interest. Draft or even complete genomes can be retrieved from metagenomics data. Therefore, strain‐level monitoring in food can be achieved. Several strain‐tracking bioinformatics approaches have been developed (Eren *et al*., 2015; Luo *et al*., 2015; Truong *et al*., 2015). The most recent, PanPhlAn, is a pangenome‐based phylogenomic analysis tool using metagenomics data to provide strain‐level microbial profiling (Scholz *et al*., 2016). This approach is used to build a pangenome of a species of interest by extracting all the genes from the available reference genomes and defining the gene family clusters. Once the gene family abundance is calculated within the metagenome, then strain‐specific genes can be identified. Therefore, it is possible to explore strain‐level diversity within the same sample and also compare foods on the basis of the different genomic signatures of strains from the target species. These strain‐level comparisons will be enormously robust and powerful and can overcome the recent disappointing attempts of strain‐level characterization of food microbes based on high‐throughput species‐specific (not rRNA‐based) amplicon sequencing (De Filippis *et al*., 2014; Ricciardi *et al*., 2016).

This high‐throughput strain‐level characterization approach can be used to investigate three different groups of food‐associated microorganisms: (i) pathogens, (ii) spoilers and (iii) fermentation players.

Genomewide strain characterization will play a key role in the identification of the pathogenic bacterial species carried by food that are responsible for food poisoning. GenomeTrakr (promoted by the FDA) is a genomic database of pathogens isolated from foodborne outbreaks. Retrieving foodborne pathogen genomes directly from metagenomics data would allow for rapid identification of the sources of foodborne outbreaks and overcome the limitations of culture‐dependent methods. These techniques will be relevant during episodes of foodborne disease outbreaks to help characterize suspected foods and contaminated food‐processing environments as well as biological specimens from the human subjects involved. Metagenomically inferred multilocus sequence typing focused on genes that encode for toxins or other pathogenic traits will be useful for wide‐scale characterizations of epidemiological interest. This approach will predict the behaviour of foodborne pathogens or strain‐dependent responses to food handling and storage conditions. It is also expected to bring forth new knowledge that will provide the basis for future risk assessments.

Similarly, *in situ* profiling of food spoiling microbes with strain‐level resolution will improve identification of their adaptation strategies in food and food‐processing environments, and the new knowledge acquired from these studies will help develop innovative preservation strategies to selectively inhibit spoilage‐associated microbial functions in food.

Lactic acid bacteria (LAB) and yeasts perform crucial functions in many fermented foods and have been extensively studied for years. They can participate in a microbiota that drives spontaneous fermentation in artisanal food making or be deliberately added to food as starter cultures. Quantitative monitoring of the key functions of strains involved in fermentation and food ripening would be very informative. The behaviour of microbial species in food is strain‐dependent and poorly predicted. A high‐throughput strain‐level characterization of food fermentative processes will allow monitoring of the succession of different strains from the same species during manufacturing, observation of shifts in strain abundance according to system variations and recognition of strains with activities relevant to specific processes. This will enable customization of strain‐level composition of microbial starters according to specific performance and needs. Moreover, when we couple strain‐level metagenomics and metatranscriptomics, we will understand how to direct and exploit this genomic potential for our research. We will better predict the microbial response to changes in food fermentation or ripening conditions, which will be useful for designing strain‐targeted process interventions to exploit microbial performances for process optimization and increased quality. Observing changes in genomic features between cohabiting strains of the same species in a food microbiome will give the unprecedented possibility to use processing conditions to modulate technologically relevant activities in specific strains. In other words, we might soon be capable of deciding the exact dynamics and conditions necessary for proteolytic or lipolytic activity to obtain a desired cheese aroma because we will know how such functions are regulated by human intervention in ripening conditions. In the context of natural fermentations, comparative genomics of strains from the metagenomes of natural starters will help us understand what raw materials and food sources yield the best possible fermentation performers, better understand the dynamics that drive the selection of specific activities, and guide the development of specific strains that deliver desirable food products. Finally, there is still hope to discover unculturable and undescribed genotypes with interesting features from natural starters.

It is time to dive into the complex metagenomes of food and obtain the best possible strain characterizations that can be used for the improvement of food quality and safety.

## Conflict of interest

The authors declare that they have no competing interest.

## References

[mbt212593-bib-0001] Cocolin, L. , and Ercolini, D. (2015) Zooming into food‐associated microbial consortia: a “cultural” evolution. Curr Opin Food Sci 2: 43–50.

[mbt212593-bib-0002] De Filippis, F. , La Storia, A. , Stellato, G. , Gatti, M. , and Ercolini, D. (2014) A selected core microbiome drives the early stages of three popular Italian cheese manufactures. PLoS One 9: e89680. 24586960 10.1371/journal.pone.0089680 PMC3933672

[mbt212593-bib-0003] De Filippis, F. , Genovese, A. , Ferranti, P. , Gilbert, J.A. , and Ercolini, D. (2016) Metatranscriptomics revels temperature‐driven functional changes in microbiome impacting cheese maturation rate. Sci Rep 6: 21871. 26911915 10.1038/srep21871 PMC4766472

[mbt212593-bib-0004] Ercolini, D. (2013) High‐throughput sequencing and metagenomics: moving forward in the culture‐independent analysis of food microbial ecology. Appl Environ Microbiol 79: 3148–3155. 23475615 10.1128/AEM.00256-13 PMC3685257

[mbt212593-bib-0005] Eren, A.M. , Esen, Ö.C. , Quince, C. , Vineis, J.H. , Morrison, H.G. , Sogin, M.L. , and Delmont, T.O. (2015) Anvi'o: an advanced analysis and visualization platform for ‘omics data. Peer J 3: e1319. 26500826 10.7717/peerj.1319 PMC4614810

[mbt212593-bib-0006] Luo, C. , Knight, R. , Sijander, H. , Knip, M. , Xavier, R.J. , and Gevers, D. (2015) ConStrains identifies microbial strains in metagenomic datasets. Nat Biotechnol 33: 1045–1052. 26344404 10.1038/nbt.3319 PMC4676274

[mbt212593-bib-0007] Parente, E. , Cocolin, L. , De Filippis, F. , Zotta, T. , Ferrocino, I. , O'Sullivan, O. , *et al* (2016) FoodMicrobionet: a database for the visualization and exploration of food bacterial communities based on network analysis. Int J Food Microbiol 219: 28–37. 26704067 10.1016/j.ijfoodmicro.2015.12.001

[mbt212593-bib-0008] Ricciardi, A. , De Filippis, F. , Zotta, T. , Facchiano, A. , Ercolini, D. , and Parente, E. (2016) Polymorphism of the phosphoserine phosphatase gene in *Streptococcus thermophilus* and its potential use for typing and monitoring of population structure. Int J Food Microbiol 236: 138–147. 27497152 10.1016/j.ijfoodmicro.2016.07.031

[mbt212593-bib-0009] Scholz, M. , Ward, D.V. , Pasolli, E. , Tolio, T. , Zolfo, M. , Asnicar, F. , *et al* (2016) Strain‐level microbial epidemiology and population genomics from shotgun metagenomics. Nat Methods 13: 435–438. 26999001 10.1038/nmeth.3802

[mbt212593-bib-0010] Truong, D.T. , Franzosa, E.A. , Tickle, T.L. , Scholz, M. , Weingart, G. , Pasolli, E. , *et al* (2015) MetaPhlAn2 for enhanced metagenomic taxonomic profiling. Nat Methods 12: 902–903. 26418763 10.1038/nmeth.3589

[mbt212593-bib-0011] Wolfe, B.E. , Button, J.E. , Santarelli, M. , and Dutton, R.J. (2014) Cheese rind communities provide tractable systems for *in situ* and *in vitro* studies of microbial diversity. Cell 158: 422–433. 25036636 10.1016/j.cell.2014.05.041 PMC4222527

